# Supplementary material for: The Role of Vitamins in Oral Health. A Systematic Review and Meta-Analysis
Source: Int J Environ Res Public Health. 2020 Feb 3;17(3):938. doi: 10.3390/ijerph17030938 (PMC7037089; doi:10.3390/ijerph17030938)
Supplement: Supplementary file 1 [file ijerph-17-00938-s001.pdf]

Table S1. List of excluded papers.

ID	*Author*	*Year*	*Sources*	*reason for exclusion*
1	Lebwohl et al.,	2019	J Am Acad Dermatol. 2019 Jan;80(1):282-285	not pertinent
2	Pola≈Ñska et al.,	2019	Int J Dermatol. 2019 Jan;58(1):108-113	not pertinent
3	Stein Gold et al.,	2018	J Drugs Dermatol. 2018 Dec 1;17(12):1290-1296.	not pertinent
4	Kakade et al.,	2018	J Dent Child (Chic). 2018 Jan 15;85(1):40-42	case study
5	Gold et al.,	2018	J Am Acad Dermatol. 2018 Aug;79(2):287-293	not pertinent
6	Gutierrez et al.,	2018	Am J Case Rep. 2018 Apr 3;19:392-396.	case study
7	Uruevitaa-Palacio et al.,	2018	BMJ Case Rep. 2018 Mar 30;2018.	not pertinent
8	Barbosa et al.,	2018	Lasers Med Sci. 2018 Aug;33(6):1255-1262.	not pertinent
9	Ingram et al.,	2018	J Dermatolog Treat. 2018 Nov;29(7):648-657	not pertinent
10	Wjcik	2018	Medicine (Baltimore). 2018 Feb;97(8):e9811.	not pertinent
11	Dobrosõ et al.,	2018	Quintessence Int. 2018;49(5):407-412.	case study
12	Cha et al:.,	2018	Osteoporos Int. 2018 Apr;29(4):987-992.	not pertinent
13	Cuny et al.,	2018	Int J Clin Pharmacol Ther. 2018 Jan;56(1):24-27	case study
14	Jung et al.,	2018	Int J Environ Res Public Health. 2018 Sep 14;15(9)	different outcome (Salivary IgA)
15	Sten Gold et al.,	2018	J Drugs Dermatol. 2018 Aug 1;17(8):863-868	not pertinent
16	Bagel et al.,	2018	J Drugs Dermatol. 2018 Aug 1;17(8):845-850.	not pertinent
17	Pariser et al,	2018	J Drugs Dermatol. 2018 Jul 1;17(7):723-726.	not pertinent
18	Dayal et al.,	2018	An Bras Dermatol. 2018 Jun;93(3):385-390	not pertinent
19	Gerdes et al.,	2017	Dermatology. 2017;233(6):425-434	not pertinent
20	Adil et al.,	2017	Acta Dermatovenerol Croat. 2017 Oct;25(3):251-253.	not pertinent
21	Rakshasbhuvankar et al.,	2017	BMC Pediatr. 2017 Dec 16;17(1):204.	not pertinent
22	Bozkaya et al.,	2017	J Cancer Res Ther. 2017 Oct-Dec;13(6):1070-1072	not pertinent
23	Kim et al.,	2017	J Stroke Cerebrovasc Dis. 2018 Mar;27(3):816-818	not pertinent
24	Pastor-Nieto et al,.	2017	Contact Dermatitis. 2017 Nov;77(5):343-345	not pertinent
25	Del Duca et al.,	2017	Int J Immunopathol Pharmacol. 2017 Dec;30(4):439-444.	not pertinent
26	Bascoul-Mollevi et al.,	2017	Eur J Cancer. 2017 Oct;84:239-249.	not pertinent
27	Hashim et al.,	2017	J Drugs Dermatol. 2017 Aug 1;16(8):747-752	not pertinent
28	Geng et al.,	2017	Clin Rheumatol. 2018 Jan;37(1):257-263.	not pertinent
29	Osawa etal.,	2017	Clin Cardiol. 2017 Oct;40(10):807-813	not pertinent
30	Saleky et al.,	2017	Cutis. 2017 Jun;99(6):431-435.	not pertinent
31	et al.,	2017	Eur J Appl Physiol. 2017 Aug;117(8):1657-1668.	not pertinent
32	Wang et al.,	2017	Dermatol Online J. 2017 May 15;23(5).	not pertinent
33	Bartley	2017	N Z Med J. 2017 Apr 28;130(1454):86-87.	not pertinent
34	Brandy-Garcia et al,	2017	Reumatol Clin. 2017 Nov - Dec;13(6):364-365	not pertinent
35	Sugarman et al.,	2017	J Drugs Dermatol. 2017 Mar 1;16(3):197-204.	not pertinent
36	Florez et al.,	2017	Nephron. 2017;136(2):111-120.	not pertinent
37	Queille-Roussel et al.,	2017	Clin Drug Investig. 2017 Apr;37(4):355-361	not pertinent
38	Erdle et al.,	2017	CMAJ. 2017 Feb 21;189(7):E275-E278.	not pertinent
39	Garcia et al,.	2017	Eur J Nutr. 2017 Sep;56(6):2081-2091	not pertinent
40	Locati et al.,	2016	Eur J Cancer. 2016 Dec;69:158-165.	not pertinent

41	Corden et al.,	2016	Clin Exp Dermatol. 2016 Dec;41(8):899-901.	not pertinent
42	Berrevoets & Bleeker-Rovers	2016	Ann Emerg Med. 2016 Sep;68(3):e65-6.	not pertinent
43	Hercogov et al.,	2016	J Biol Regul Homeost Agents. 2016 Apr-Jun;30(2 Suppl 3):77-81.	not pertinent
44	Louis et al.,	2016	Eur J Appl Physiol. 2016 Oct;116(10):1941-54.	not pertinent
45	Janssens et al.,	2016	Radiother Oncol. 2016 May;119(2):213-20.	not pertinent
46	Kato & Yamamoto	2016	J Dermatol. 2016 Oct;43(10):1238-1239	not pertinent
47	Aggarwal et al.,	2016	J Dermatolog Treat. 2016 Nov;27(6):546-551	not pertinent
48	Snape et al.,	2016	Br J Dermatol. 2016 Sep;175(3):479-86	not pertinent
49	Luna et al.,	2016	Pediatr Dermatol. 2016 Mar-Apr;33(2):e65-8.	not pertinent
50	Youssef et al.,	2016	Saudi J Kidney Dis Transpl. 2016 Jan;27(1):144-6	not pertinent
51	Oaks et al.,	2016	J Nutr. 2016 Feb;146(2):343-52.	not pertinent
52	Chandra et al,	2016	Cutis. 2015 Dec;96(6):E1-2.	not pertinent
53	Türkmen et al.,	2016	Niger J Clin Pract. 2016 Jan-Feb;19(1):91-8.	in vitro
54	Ronsein et al.,	2016	Arterioscler Thromb Vasc Biol. 2016 Feb;36(2):404-11	not pertinent
55	Gracia et al.,	2016	Clin J Am Soc Nephrol. 2016 Feb 5;11(2):287-96	not pertinent
56	Yiu et al.,	2016	Clin Exp Dermatol. 2016 Jun;41(4):407-9	not pertinent
57	Ranchordas et al.,	2016	Int J Sport Nutr Exerc Metab. 2016 Apr;26(2):185-91	not pertinent
58	He et al.,	2016	J Sports Sci. 2016;34(1):67-74	not pertinent
59	Levin et al.,	2015	J Dermatolog Treat. 2015 Feb;26(1):16-8	not pertinent
60	Segal D	2015	Dermatol Online J. 2013 May 15;19(5):18175.	not pertinent
61	Golusin et al.,	2015	Vojnosanit Pregl. 2015 Nov;72(11):1010-7	not pertinent
62	Stewart et al.,	2015	BMC Pregnancy Childbirth. 2015 Dec 22;15:346.	not pertinent
63	Angsanuntsukh et al.,	2015	J Med Assoc Thai. 2015 Sep;98 Suppl 8:S95-101	not pertinent
64	Tomar & Aggarwal	2015	Indian Heart J. 2015 Sep-Oct;67(5):491-2	not pertinent
65	Kotsia et al.,	2015	J Invasive Cardiol. 2015 Oct;27(10):E204-10	not pertinent
66	Hammami et al.,	2015	BMC Cancer. 2015 Sep 9;15:624	not pertinent
67	Amato et al.,	2015	Drug Des Devel Ther. 2015 Jul 23;9:3877-84	not pertinent
68	AlJehani	2015	J Contemp Dent Pract. 2015 Apr 1;16(4):319-21	not pertinent
69	Sheel et al.,	2015	BMJ Case Rep. 2015 Jun 8;2015	not pertinent
70	Fernandez et al.,	2015	Pediatrics. 2015 Jun;135(6):e1518-23	not pertinent
71	Dyring-Andersen et al.,	2015	Scand J Immunol. 2015 Jul;82(1):84-91	not pertinent
72	Schulze-Späte et al.,	2015	J Clin Endocrinol Metab. 2015 Jun;100(6):2425-33	not pertinent
73	Fakuda et al.,	2015	PLoS One. 2015 Mar 6;10(3):e0119578	not pertinent
74	Abu Hilal & Ho	2015	Pediatr Dermatol. 2015 May-Jun;32(3):377-80	not pertinent
75	Queille-Roussel et al.,	2015	Clin Drug Investig. 2015 Apr;35(4):239-45	not pertinent
76	Papp et al.,	2015	Br J Dermatol. 2015 Sep;173(3):767-76	not pertinent
77	Da Boit e al.,m	2015	Int J Sports Med. 2015 May;36(5):426-30	not pertinent
78	Hallas et al.,	2015	J Pediatr Health Care. 2015 May-Jun;29(3):283-8	not pertinent
79	Hendriks et al.,	2015	Skin Res Technol. 2015 Aug;21(3):340-5	not pertinent
80	Graziani et al.,	2015	Oral Health Prev Dent. 2015;13(2):101-11	not pertinent
81	Garaiova et al.,	2015	Eur J Clin Nutr. 2015 Mar;69(3):373-9	not pertinent
82	Jaatinen et al.,	2014	Int J Food Sci Nutr. 2014 Jun;65(4):507-14	not pertinent
83	Souza et al.,	2014	J Appl Oral Sci. 2013 Nov-Dec;21(6):601-6	case report
84	Hirohata A	2014	EuroIntervention. 2014 Jan 22;9(9):1050-6. doi: 10.4244/EIJV9I9A178	not pertinent
85	Kluesner NH	2014	J Emerg Med. 2014 Apr;46(4):530-2. doi: 10.1016/j.jemermed.2013.09.027. Epub 2014	not pertinent
86	Bryce	2014	J R Nav Med Serv. 2014;100(3):328-32	case report

87	Malakouti M	2014	Dermatol Online J. 2014 Dec 16;21(3). pii: 13030/qt76m221mt	not pertinent
88	Kantaputra PN(	2014	Am J Med Genet A. 2014 Jan;164A(1):1-9. doi: 10.1002/ajmg.a.36187. Epub 2013 Nov	case report
89	Ozturk U	2014	Ir J Med Sci. 2014 Sep;183(3):449-53. doi: 10.1007/s11845-013-1036-5. Epub 2013	not pertinent
90	Sandoval et al.,	2014	Cutis. 2014 Dec;94(6):304-9.	not pertinent
91	Shoor et al.,	2014	BMJ Case Rep. 2014 Nov 26;2014	not pertinent
92	El Ati et al.,	2014	Saudi J Kidney Dis Transpl. 2014 Sep;25(5):1072-7	not pertinent
93	Gooderham et al.,	2014	Br J Dermatol. 2014 Dec;171(6):1470-7	not pertinent
94	Zipursky et al.,	2014	BMJ Case Rep. 2014 May 23;2014	not pertinent
95	Davit-Béal et al.,	2014	Pediatrics. 2014 Apr;133(4):e1077-81	case study
96	van Geel et al.,	2014	Br J Dermatol. 2014 Aug;171(2):363-9	not pertinent
97	Betriu et al.,	2014	Nephrol Dial Transplant. 2014 Jul;29(7):1415-22	not pertinent
98	Mazzotta & Caragiuli	2014	Am J Ophthalmol. 2014 Jun;157(6):1156-62	not pertinent
99	Paulis G	2013	Inflamm Allergy Drug Targets. 2013 Dec;12(6):403-9	not pertinent
100	Camfield DA	2013	Nutrients. 2013 Nov 11;5(11):4429-50. doi: 10.3390/nu5114429	different outcome
101	Ortonne JP	2013	J Eur Acad Dermatol Venereol. 2014 Sep;28(9):1226-34. doi: 10.1111/jdv.12270.	not pertinent
102	Xiao Y	2013	PLoS One. 2013 Aug 28;8(8):e72545. doi: 10.1371/journal.pone.0072545. eCollection	not pertinent
103	Singh P	2013	J Drugs Dermatol. 2013 Aug;12(8):868-73	not pertinent
104	Lv∏land KH(	2013	PLoS One. 2013 Jul 25;8(7):e70101. doi: 10.1371/journal.pone.0070101. Print 2013.	not pertinent
105	Turner MJ	2013	Pediatr Dermatol. 2013 Sep-Oct;30(5):574-8. doi: 10.1111/pde.12187. Epub 2013 Jul	not pertinent
106	Sibley CT	2013	. Heart. 2013 Nov;99(22):1675-80. doi: 10.1136/heartjnl-2013-303926. Epub 2013 Jul	not pertinent
107	Patel D	2013	Clin Cardiol. 2013 Sep;36(9):535-41. doi: 10.1002/clc.22155. Epub 2013 Jun 10	not pertinent
108	Sarkar S	2013	J Pediatr Endocrinol Metab. 2013;26(9-10):941-3. doi: 10.1515/jpem-2013-0137	not pertinent
109	Troeltzsch et al.,	2013	Oral Surg Oral Med Oral Pathol Oral Radiol. 2013 May;115(5):e28-33.	not pertinent
110	Strippoli et al.,	2013	BMC Nephrol. 2013 Apr 19;14:90	different outcome
111	Feldam et al.,	2013	J Drugs Dermatol. 2013 Mar;12(3):300-6.	not pertinent
112	Patel et al.,	2013	Am J Cardiol. 2013 Jun 15;111(12):1681-7	not pertinent
113	Noble et al.,	2013	Psychosomatics. 2013 May-Jun;54(3):277-83	not pertinent
114	Ahmadi et al.,	2013	Int J Cardiol. 2013 Oct 3;168(3):2310-4	not pertinent
115	Rumsey and Rosenberg	2013	J Rheumatol. 2013 Feb;40(2):201-2	not pertinent
116	Paulis et al.,	2013	Andrology. 2013 Jan;1(1):120-8	not pertinent
117	Phan et al.,	2013	Am J Cardiol. 2013 Feb 1;111(3):352-5	not pertinent
118	Paulis et al.,	2013	Inflamm Allergy Drug Targets. 2013 Feb;12(1):61-7	not pertinent
119	Larijani et al.,	2013	Nutrition. 2013 Jan;29(1):71-5	not pertinent
120	Strohal et al.,	2013	J Dermatolog Treat. 2013 Jun;24(3):169-78	not pertinent
121	Greenberger et al.,	2012	J Am Coll Nutr. 2012 Oct;31(5):320-6.	not pertinent
122	Otsuki et al.,	2012	Nutr J. 2012 Dec 11;11:103	different oucome (IgA)
123	Tirado-Sancez and Ponce-Olivera	2012	Cutis. 2012 Sep;90(3):140-4.	not pertinent
124	Liu et al.,	2012	J Clin Neurosci. 2012 Dec;19(12):1689-94	not pertinent
125	Deyb et al.,	2012	Klin Padiatr. 2012 Nov;224(7):448-52	not pertinent

126	Bernardino et al.,	2012	BMJ Case Rep. 2012 Sep 17;2012	not pertinent
127	Malhotra et al.,	2012	J Dent Hyg. 2012 Summer;86(3):195-203	not pertinent
128	Queille-Roussel et al.,	2012	Clin Drug Investig. 2012 Sep 1;32(9):613-9	not pertinent
129	Sehgal et al.,	2012	Skinmed. 2012 May-Jun;10(3):183-4.	not pertinent
130	Bendick and Mey	2012	Lancet. 2012 Jun 23;379(9834):2400	not pertinent
1301	Garcia et al.,	2012	Eur J Esthet Dent. 2012 Summer;7(2):154-62.	not pertinent
132	Feldman et al.,	2012	Am J Clin Dermatol. 2012 Aug 1;13(4):261-71.	not pertinent
133	Ozkan et al.,	2012	Int J Dermatol. 2012 May;51(5):609-13	not pertinent
134	Rogalski et al.,	2012	Int J Hyperthermia. 2012;28(2):184-90	not pertinent
135	Punwani et al.,	2012	J Am Acad Dermatol. 2012 Oct;67(4):658-64	not pertinent
136	Imafuku et al.,	2012	J Dermatol. 2012 Mar;39(3):275-7	not pertinent
137	McKenna ety al.,	2012	Gerodontology. 2012 Jun;29(2):e883-90	different outcome
138	Katrancƒ et al.,	2012	Eur J Oncol Nurs. 2012 Sep;16(4):339-44	not pertinent
139	Vena et al.,	2012	J Dermatolog Treat. 2012 Aug;23(4):255-60	not pertinent
140	Feller et a.,	2012	AIDS Res Hum Retroviruses. 2012 Apr;28(4):346-51	not pertinent
141	Scarmucci et al.,	2011	Braz Dent J. 2011;22(6):473-8	not pertinent
142	Fridlington et al.,	2011	Dermatol Online J. 2011 Nov 15;17(11):2	not pertinent
143	Fisekcioglu	2011	J Can Dent Assoc. 2011;77:b148	not pertinent
144	Hudson et al.,	2011	Cutis. 2011 Oct;88(4):201-7	not pertinent
145	Otsuki et al.,	2011	Nutr J. 2011 Sep 9;10:91	different oucome (IgA)
146	Menter et al.,	2011	Cutis. 2011 Jul;88(1):46-51	not pertinent
147	Rodrigues et al.,	2011	Oper Dent. 2011 Sep-Oct;36(5):537-44	not pertinent
148	Kircik	2011	J Drugs Dermatol. 2011 Aug;10(8):878-82	not pertinent
149	Calzavara-Pinton	2011	G Ital Dermatol Venereol. 2011 Aug;146(4):295-9	not pertinent
150	Dill et al.,	2011	Mol Genet Metab. 2011 Nov;104(3):362-8	not pertinent
151	Magliocca et al.,	2011	J Oral Maxillofac Surg. 2011 Oct;69(10):2592-4	not pertinent
152	Nieman et al.,	2011	Nutr J. 2011 Apr 21;10:36	not pertinent
153	Jain et al.,	2011	Musculoskelet Surg. 2011 Dec;95(3):265-8	not pertinent
154	Kunt et al.,	2011	Acta Odontol Scand. 2011 Sep;69(5):287-91	not pertinent
155	Ryo et al.,	2011	Clin Biochem. 2011 Jun;44(8-9):669-74	not pertinent
156	Gracia et al.,	2011	Int J Dent Hyg. 2011 Nov;9(4):266-73	not pertinent
157	Prignano et al.,	2011	G Ital Dermatol Venereol. 2011 Feb;146(1):47-52.	not pertinent
158	Brodell et al.,	2011	J Drugs Dermatol. 2011 Feb;10(2):158-64	not pertinent
159	Peeters et al.,	2011	Int Arch Allergy Immunol. 2011;155(1):23-30	not pertinent
160	Singer	2011	Nephrol Dial Transplant. 2011 Feb;26(2):614-20	not pertinent
161	Resic et al.,	2011	Int Urol Nephrol. 2011 Jun;43(2):575-80	not pertinent
162	Bacci et al.,	2010	Pediatr Dent. 2010 Nov-Dec;32(7):536-8	not pertinent
163	Moosavi et al.,	2010	J Contemp Dent Pract. 2010 Dec 1;11(6):E033-40	not pertinent
164	Filaire et al.,	2010	Int J Sport Nutr Exerc Metab. 2010 Dec;20(6):496-506	not pertinent
165	Damm	2010	Gen Dent. 2010 Nov-Dec;58(6):538, 340	different outcome
166	Bashutski et al.,	2010	N Engl J Med. 2010 Dec 16;363(25):2396-405	different outcome
167	Khoroushi et al.,	2010	Oper Dent. 2010 Sep-Oct;35(5):530-7	not pertinent
168	Cavalcanti et al.,	2010	Eur Arch Paediatr Dent. 2010 Oct;11(5):253-5	not pertinent
169	Stănescu et al.,	2010	Rom J Morphol Embryol. 2010;51(3):559-63	not pertinent
170	Safarinejad	2010	Int J Impot Res. 2010 Sep-Oct;22(5):298-309	not pertinent
171	Osmancevic et al.,	2010	J Photochem Photobiol B. 2010 Nov 3;101(2):117-23	not pertinent
172	Smith et al.,	2010	Am J Dermatopathol. 2010 Dec;32(8):828, 850.	not pertinent
173	Manson et al.,	2010	Menopause. 2010 Jul;17(4):683-91	not pertinent

174	Alora-Palli et al.,	2010	Am J Clin Dermatol. 2010;11(4):275-83	not pertinent
175	Brouda et al.,	2010	Cutis. 2010 Apr;85(4):214-20	not pertinent
176	D'Alessandro et al.,	2010	Minerva Stomatol. 2010 Mar;59(3):129-37	not pertinent
177	Saxena and Dogra	2010	Eur J Dermatol. 2010 May-Jun;20(3):329-33	not pertinent
178	Liu et al.,	2010	J Nucl Med. 2010 Apr;51(4):618-23	different outcome
179	Bailey and Whitehair	2010	Am Fam Physician. 2010 Mar 1;81(5):596.	not pertinent
180	van der Velden et al.	2010	J Dermatolog Treat. 2010 Jan;21(1):13-22	not pertinent
181	Kumar et al.,	2010	Clin Exp Dermatol. 2010 Jul;35(5):482-6	not pertinent
182	Kvist et al.	2009	J Transl Med. 2009 Dec 17;7:107	not pertinent
183	Velez et al.,	2009	J Clin Pediatr Dent. 2009 Fall;34(1):67-9	not pertinent
184	Rosina et al.,	2009	Skin Res Technol. 2009 May;15(2):135-8	not pertinent
185	Chappell et al.,	2009	J Drugs Dermatol. 2009 Jun;8(6):573-6	not pertinent
186	Lebwohl et al.,	2009	Cutis. 2009 Apr;83(4):205-12	not pertinent
187	Zamora and Males	2009	Cornea. 2009 May;28(4):474-6	not pertinent
188	Motamedi et al.,	2009	Dent Today. 2009 Feb;28(2):102, 104-5	not pertinent
189	Schneider et al.,	2009	Invest New Drugs. 2009 Dec;27(6):571-8	not pertinent
190	Menter et al.,	2009	J Drugs Dermatol. 2009 Jan;8(1):52-7	not pertinent
191	Alexandrescu et al.,	2009	Clin Exp Dermatol. 2009 Oct;34(7):811-4	not pertinent
192	Nenseter et al.,	2009	Stroke. 2009 Jan;40(1):241-7	not pertinent
193	Mallett et al.,	2009	Ultrasound Med Biol. 2009 Mar;35(3):367-75.	not pertinent
194	Carrillo et al.,	2008	Int J Sports Physiol Perform. 2008 Dec;3(4):516-30.	different oucome (IgA)
195	Li et al.,	2008	Am J Otolaryngol. 2008 Nov-Dec;29(6):426-8	not pertinent
196	Bansal and Tewari	2008	Int Endod J. 2008 Nov;41(11):950-7	not pertinent
197	van Linger et al.,	2008	Eur J Dermatol. 2008 Nov-Dec;18(6):671-6	not pertinent
198	Held et al.,	2008	Vasc Med. 2008 Nov;13(4):245-53	not pertinent
199	Léger	2008	Can Fam Physician. 2008 Oct;54(10):1403-6	not pertinent
200	de Silva et al.,	2008	Med Oral Patol Oral Cir Bucal. 2008 Sep 1;13(9):E559-62	not pertinent
201	Muller et al.,	2008	Braz Dent J. 2008;19(2):145-50	not pertinent
202	Padmavathy et al.,	2008	Indian J Tuberc. 2008 Apr;55(2):97-9	not pertinent
203	Cortellini et al.,	2008	J Clin Periodontol. 2008 Jul;35(7):614-20	not pertinent
204	Zare et al.,	2008	Eur Neurol. 2008;59(6):299-301	different oucome (IgA)
205	Disma et al.,	2008	Paediatr Anaesth. 2008 Jul;18(7):672-3	not pertinent
206	Vissers et al.,	2008	Br J Dermatol. 2008 Apr;158(4):705-12	not pertinent
207	Chládek et al.,	2008	Eur J Clin Pharmacol. 2008 Apr;64(4):347-55	not pertinent
208	de Korte et al.,	2008	Br J Dermatol. 2008 Feb;158(2):375-81	not pertinent
209	Saito et al.,	2008	Brain Dev. 2008 Mar;30(3):221-5	not pertinent
210	Lebwohl and Colón	2007	Cutis. 2007 Nov;80(5 Suppl):29-40	not pertinent
211	Matsuo et al.,	2007	Am Heart J. 2007 Nov;154(5):994.e1-6	not pertinent
212	Liao et al.,	2007	Br J Dermatol. 2007 Nov;157(5):1005-12	not pertinent
213	Manning et al.,	2007	Scand J Gastroenterol. 2007 Dec;42(12):1413-21	not pertinent
214	Ezguerra et al.,	2007	Acta Derm Venereol. 2007;87(5):449-50	not pertinent
215	Safarinejad et al.,	2007	J Urol. 2007 Oct;178(4 Pt 1):1398-403	not pertinent
216	Lebwohl et al.,	2007	J Drugs Dermatol. 2007 Apr;6(4):428-35	not pertinent
217	Rodrigues Santos et al.,	2007	Spec Care Dentist. 2007 May-Jun;27(3):108-11	not pertinent
218	Duggan et al.,	2007	N Engl J Med. 2007 Jul 26;357(4):392-400	not pertinent
219	Zhao et al.,	2007	Am Heart J. 2007 Aug;154(2):239-46	not pertinent
220	Helfrich et al.,	2007	Br J Dermatol. 2007 Aug;157(2):369-74	not pertinent
221	Friedman K	2007	N Y State Dent J. 2007 Apr;73(3):34-7.	not pertinent
222	Burk CJ	2007	Am J Clin Dermatol. 2007;8(2):103-6.	case report
223	Zhu X	2007	J Eur Acad Dermatol Venereol. 2007 Apr;21(4):466-72.	not pertinent

224	Chang CW	2007	Dig Dis Sci. 2007 May;52(5):1259-61. Epub 2007 Mar 20.	case report
225	Angelo JS	2007	Indian J Dermatol Venereol Leprol. 2007 Jan-Feb;73(1):65	not pertinent
226	Nagpal R	2007	Oper Dent. 2007 Jan-Feb;32(1):16-23.	trial in lab
227	K√∂rver JE	2007	Br J Dermatol. 2007 Jan;156(1):130-7.	not pertinent
228	Zaromb A	2006	J Periodontol. 2006 Nov;77(11):1921-6.	case report
229	Koo J.	2006	J Am Acad Dermatol. 2006 Oct;55(4):637-41.	not pertinent
230	van de Kerkhof PC	2006	Br J Dermatol. 2006 Oct;155(4):800-7.	not pertinent
231	Erceg A	2006	Br J Dermatol. 2006 Jul;155(1):110-4.	not pertinent
232	E Adişen	2006	J Eur Acad Dermatol Venereol. 2006 May;20(5):527-33.	not pertinent
233	Ortonne JP	2006	Acta Derm Venereol. 2006;86(1):29-33	not pertinent
234	Inal T	2006	Urology. 2006 May;67(5):1038-42. Epub 2006 Apr 11	not pertinent
235	Veraldi S	2006	Dermatology. 2006;212(3):235-7	not pertinent
236	Batra P	2006	J Can Dent Assoc. 2006 Feb;72(1):69-72.	case report
237	Dawson SJ	2006	Nat Clin Pract Oncol. 2006 Feb;3(2):104-7.	not pertinent
238	Roussaki-Schulze AV	2005	Drugs Exp Clin Res. 2005;31(5-6):169-74	not pertinent
239	Brazzelli V	2005	Int J Immunopathol Pharmacol. 2005 Oct-Dec;18(4):755-60	not pertinent
240	Poujol S	2005	Cancer Chemother Pharmacol. 2006 Sep;58(3):292-305. Epub 2005 Dec 21.	not pertinent
241	Halligan TJ	2005	Oral Surg Oral Med Oral Pathol Oral Radiol Endod. 2005 Dec;100(6):688-92. Epub	case report
242	Carboni I	2005	J Eur Acad Dermatol Venereol. 2005 Nov;19 Suppl 3:11-3.	not pertinent
243	Chen CM	2005	World J Gastroenterol. 2005 Oct 7;11(37):5777-81.	not pertinent
244	Dervis E	2005	Pediatr Dermatol. 2005 Sep-Oct;22(5):436-9	not pertinent
245	Tzung TY	2005	Acta Derm Venereol. 2005;85(3):236-9	not pertinent
246	M√§kinen KK	2005	Caries Res. 2005 May-Jun;39(3):207-15.	case study
247	Wolf NI	2005	Neurology. 2005 Apr 26;64(8):1461-4.	case report
248	S√°nchez AR	2004	J Periodontol. 2004 Dec;75(12):1714-9	case report
249	Claro JA	2004	Int Braz J Urol. 2004 May-Jun;30(3):199-204;	not pertinent
250	Prasad VN	2004	J Indian Soc Pedod Prev Dent. 2004 Jun;22(2):82-91	case study
251	Hildebolt CF	2004	J Periodontol. 2004 Jun;75(6):811-6	case study
252	Nieman DC	2004	Med Sci Sports Exerc. 2004 Aug;36(8):1328-35.	not pertinent
253	Durakovic C	2004	Br J Dermatol. 2004 Jul;151(1):190-5	not pertinent
254	Franssen ME	2004	Acta Derm Venereol. 2004;84(3):195-200	not pertinent
255	Jung J	2004	Dermatol Surg. 2004 Jun;30(6):931-3	not pertinent
256	Apisarnthanarax N	2004	J Am Acad Dermatol. 2004 Apr;50(4):600-7	not pertinent
257	Vissers WH	2004	Exp Dermatol. 2004 Feb;13(2):106-12	not pertinent
258	Micheletta F	2004	Arterioscler Thromb Vasc Biol. 2004 Jan;24(1):136-40. Epub 2003 Oct 30	not pertinent
259	Cao L	2003	J Adhes Dent. 2003 Summer;5(2):139-44	lab trial
260	Tay YK	2003	J Dermatolog Treat. 2003 Dec;14(4):219-21	not pertinent
261	Spence JD	2003	Clin Chem Lab Med. 2003 Nov;41(11):1498-504	not pertinent
262	Kaya AD	2003	Oper Dent. 2003 Nov-Dec;28(6):825-9	lab trial
263	Sharma V	2003	Int J Dermatol. 2003 Oct;42(10):834-8	not pertinent
264	Katoh N	2003	Eur J Dermatol. 2003 Jul-Aug;13(4):382-4.	not pertinent
265	Holick MF	2003	Br J Dermatol. 2003 Aug;149(2):370-6.	not pertinent
266	Tzaneva S	2003	Br J Dermatol. 2003 Aug;149(2):350-3.	not pertinent
267	Maekawa S	2003	Resuscitation. 2003 Aug;58(2):227-30	not pertinent
268	Lahfa M	2003	Eur J Dermatol. 2003 May-Jun;13(3):261-5	not pertinent
269	Zambrano M	2003	Oral Surg Oral Med Oral Pathol Oral Radiol Endod. 2003 Jun;95(6):705-9.	case report

270	Uhoda I	2003	Dermatology. 2003;206(4):366-9	not pertinent
271	Hofmann UB	2003	Br J Dermatol. 2003 Apr;148(4):779-83	not pertinent
272	Camarasa JM	2003	J Dermatolog Treat. 2003 Jan;14(1):8-13	not pertinent
273	Cherian T	2003	Vaccine. 2003 Jun 2;21(19-20):2418-20	not pertinent
274	Weinstein GD	2003	J Am Acad Dermatol. 2003 May;48(5):760-7	not pertinent
275	Kuenzli S	2003	Dermatology. 2003;206(3):252-6	not pertinent
276	Prieto Castro RM	2003	BJU Int. 2003 Apr;91(6):522-4.	not pertinent
277	Ortonne JP	2003	Br J Dermatol. 2003 Feb;148(2):326-33	not pertinent
278	Hecht HS	2003	Am J Cardiol. 2003 Feb 1;91(3):348-51	not pertinent
279	Andreassi L	2003	Br J Dermatol. 2003 Jan;148(1):134-8	not pertinent
280	Mascarv≥ JM	2002	Cutis. 2002 Nov;70(5 Suppl):13-5	not pertinent
281	Bunout D	2002	JPEN J Parenter Enteral Nutr. 2002 Nov-Dec;26(6):372-6	not pertinent
282	Tzaneva S	2002	Br J Dermatol. 2002 Oct;147(4):748-53	not pertinent
283	Erdogru T	2002	Asian J Androl. 2002 Sep;4(3):187-90	not pertinent
284	Monastirli A	2002	Skin Pharmacol Appl Skin Physiol. 2002 Jul-Aug;15(4):246-51	not pertinent
285	Shroff DV	2002	Pediatr Dent. 2002 Jul-Aug;24(4):347-9	case report
286	Gollnick H	2002	Dermatology. 2002;205(1):46-53	not pertinent
287	Lambert J	2002	Dermatology. 2002;204(4):321-4	not pertinent
288	Bowman PH	2002	J Am Acad Dermatol. 2002 Jun;46(6):907-13.	not pertinent
289	Stvºcker M	2002	Skin Res Technol. 2002 May;8(2):133-40	not pertinent
290	Marshall R	2002	Aust Dent J. 2002 Mar;47(1):82; author reply 82-3.	?
291	Allen F	2002	Clin Oral Implants Res. 2002 Jun;13(3):320-6	not pertinent
292	Green L	2002	J Cutan Med Surg. 2002 Mar-Apr;6(2):95-102. Epub 2002 Feb 13	not pertinent
293	van de Kerkhof PC	2002	Br J Dermatol. 2002 Mar;146(3):414-22	not pertinent
294	Fang JC	2002	Lancet. 2002 Mar 30;359(9312):1108-13	not pertinent
295	Muv±oz CA	2002	Compend Contin Educ Dent. 2001 May;22(5):425-8, 430, 432 passim; quiz 440	case study
296	Lee HS	2002	Can J Cardiol. 2002 Mar;18(3):271-5	not pertinent
297	Brody S	2002	Psychopharmacology (Berl). 2002 Jan;159(3):319-24. Epub 2001 Nov 20	rtc animals
298	Morrone LF	2002	J Nephrol. 2001 Sep-Oct;14(5):415-9.	not pertinent
299	Stvºcker M	2001	Dermatology. 2001;203(2):141-7	not pertinent
300	Kaur I	2001	J Dermatol. 2001 Aug;28(8):448-50	not pertinent
301	Novak TE	2001	J La State Med Soc. 2001 Jul;153(7):358-63	not pertinent
302	Galderma R & D	2001	Br J Dermatol. 2001 Apr;144 Suppl 58:3-10	not pertinent
303	Kowalzick L	2001	Br J Dermatol. 2001 Apr;144 Suppl 58:21-5	not pertinent
304	Gerritsen MJ	2001	Br J Dermatol. 2001 Apr;144 Suppl 58:17-9	not pertinent
305	Langner A	2001	Br J Dermatol. 2001 Apr;144 Suppl 58:11-6	not pertinent
306	Koo JY	2001	Int J Dermatol. 2001 Mar;40(3):210-2	not pertinent
307	Queille-Roussel	2001	Eur J Dermatol. 2001 May-Jun;11(3):219-24	not pertinent
308	Durakovic C	2001	Br J Dermatol. 2001 Mar;144(3):500-6	not pertinent
309	Ring J	2001	Br J Dermatol. 2001 Mar;144(3):495-9	not pertinent
310	Salmhofer W	2001	Acta Derm Venereol Suppl (Stockh). 2000;(211):5-8	not pertinent
311	Schmugge M	2001	Eur J Pediatr. 2001 Jan;160(1):43-6	case report
312	Spence JD	2001	Neuroepidemiology. 2001 Feb;20(1):16-25	not pertinent
313	Tardif JC	2001	Can J Cardiol. 2001 Jan;17(1):49-55	case study
314	Tanghetti EA	2000	Cutis. 2000 Dec;66(6 Suppl):4-11	not pertinent
315	Coynik D	2000	Cutis. 2000 Dec;66(6 Suppl):19-24	not pertinent
316	Tanghetti EA	2000	Cutis. 2000 Dec;66(6 Suppl):12-8	not pertinent
317	Jeong MH	2000	Jpn Circ J. 2000 Nov;64(11):897-900	not pertinent
318	Guenther LC	2000	Clin Ther. 2000 Oct;22(10):1225-38	not pertinent
319	Halpner AD	2000	J Womens Health Gend Based Med. 2000 Nov;9(9):995-8	not pertinent

320	Hutchinson PE	2000	Dermatology. 2000;201(2):139-45	not pertinent
321	Koo JY	2000	J Am Acad Dermatol. 2000 Nov;43(5 Pt 1):821-8	not pertinent
322	Wilkins K	2000	J Cutan Med Surg. 2000 Jul;4(3):164-8	not pertinent
323	Murayama T	2000	Oral Surg Oral Med Oral Pathol Oral Radiol Endod. 2000 Sep;90(3):310-	case report
324	Aine L	2000	J Oral Pathol Med. 2000 Sep;29(8):403-9	case study
325	Mader I	2000	AJNR Am J Neuroradiol. 2000 Aug;21(7):1220-7	not pertinent
326	Levin NA	2000	Cutis. 2000 Jul;66(1):39-44	case report
327	Lebwohl M	2000	J Am Acad Dermatol. 2000 Aug;43(2 Pt 3):S43-6	not pertinent
328	Krause I	2000	Pediatr Nephrol. 2000 Jun;14(6):499-501	case report
329	Singh S	2000	J Am Acad Dermatol. 2000 Jul;43(1 Pt 1):61-5	not pertinent
330	Ellis CN	2000	Arch Dermatol. 2000 May;136(5):609-16	not pertinent
331	Leivo T	2000	Br J Dermatol. 2000 May;142(5):991-1002	not pertinent
332	Hackam DG	2000	Am J Hypertens. 2000 Jan;13(1 Pt 1):105-10	not pertinent
333	Behrens S	2000	J Am Acad Dermatol. 2000 Mar;42(3):493-5	not pertinent
334	Castelijns FA	2000	Dermatology. 2000;200(1):25-30	not pertinent

Table S2. Quality assessment. **Table S2.** Quality assessment.

	CRITERIA															
Authors	**1**	**2**	**3**	**4**	**5**	**6**	**7**	**8**	**9**	**10**	**11**	**12**	13	14	Final	Grading
Syed et al.,	1	N/A	N/A	N/A	1	1	1	1	N/A	N/A	0	0	0	0	5	Poor
Gyll et al.,	1	1	0	1	1	1	1	1	1	1	1	0	0	1	11	Good
Li et al.,	1	1	1	1	1	1	1	1	1	1	1	1	0	1	13	Good
kim et al.,	0	1	0	1	1	1	1	1	0	1	0	1	0	1	10	Good
Wójcik et al.,	1	N/A	N/A	0	0	0	1	0	N/A	1	1	1	N/A	1	6	Poor
Seminario et al.,	1	1	0	1	1	1	1	1	0	1	1	1	0	1	11	Good
van der Tas et al.,	1	1	N/A	N/A	1	1	1	1	N/A	1	1	1	N/A	1	10	Good
Balci Yuce et al.,	1	1	1	1	1	1	1	1	0	1	1	1	0	1	12	Good
Deepti et al.,	1	1	1	1	1	1	1	1	0	1	1	1	0	1	12	Good
Kühnisch et al.,	1	1	N/A	1	1	1	1	1	1	1	1	1	0	1	12	Good
Reed et al.,	1	1	N/A	1	1	N/A	1	N/A	1	1	1	1	N/A	1	10	Good
Abreu et al.	1	1	1	1	1	0	0	N/A	0	1	1	1	0	1	9	Fair
Adegboye et al.,	1	1	N/A	1	1	0	0	1	1	1	1	1	0	1	10	Good
Gümüş et al.,	1	1	N/A	1	1	0	0	1	1	1	1	1	0	1	10	Good
Pavlesen et al.,	1	1	1	1	1	1	1	1	1	1	1	1	0	1	13	Good
Woelber et al.,	1	1	1	1	1	0	1	1	1	0	1	1	1	1	12	Good
Kühnisch et al.,	1	1	N/A	1	1	0	0	1	1	0	1	1	1	1	11	Good
Shimabukuro et al.,	1	1	1	1	1	0	0	1	1	0	1	1	1	1	12	Good
Dudding et al.,	1	0	1	1	1	0	0	1	1	1	1	1	1	1	11	Good
Lee et al.,	1	1	1	1	1	0	1	1	1	1	1	1	N/A	1	12	Good
Tanaka et al.,	1	1	1	1	1	1	1	1	1	1	1	1	0	1	13	Good
Schroth et al.,	1	0	1	0	1	0	1	1	0	1	0	1	0	1	8	Fair
Singh et al.,	1	1	1	1	1	0	0	1	1	1	1	1	0	1	11	Good
Jimenez et al.,	1	1	1	0	1	0	0	1	N/A	N/A	1	1	1	1	10	Good
Alshouibi et al.,.	1	1	1	0	1	0	1	1	1	N/A	1	1	0	1	10	Good
Gokhale et al.,	1	1	0	0	1	0	1	0	0	0	1	1	0	1	7	Fair
Hiremath et al.,	1	1	1	1	1	0	1	1	1	1	1	1	0	1	12	Good
Iwasaki et al.,	1	1	1	1	1	1	1	1	1	1	1	1	0	1	13	Good
Millen et al.,	1	1	1	1	1	1	1	1	1	1	1	1	0	1	13	Good
Teles et .,	1	1	N/A	N/A	1	1	1	N/A	1	N/A	1	1	0	1	9	Fair
Schroth et al.,	1	1	1	1	1	0	1	1	1	1	1	1	1	0	12	Good
Zhang et al.,	1	1	N/A	N/A	1	1	1	N/A	1	N/A	1	1	1	0	9	Fair
Aidi	1	1	N/A	1	1	1	1	N/A	1	1	1	1	0	1	11	Good
Araya et al.,	1	1	1	1	0	1	1	N/A	1	1	1	1	1	1	12	Fair
Bashutski et al.,	1	1	N/A	1	1	1	1	1	1	1	1	1	0	1	12	Good
Boggess et al.,	1	1	N/A	1	0	1	1	N/A	1	1	1	1	0	1	10	Good
Harpenau et al.,	1	1	N/A	0	1	N/A	0	0	1	0	0	0	0	1	5	Poor
Liu et al.,	1	1	0	0	1	N/A	1	0	1	0	1	0	1	1	8	Fair
Willershausen et al.,	1	1	N/A	N/A	1	1	1	0	1	0	1	1	0	1	9	Fair
Abou Sulaiman, et al.,	1	1	N/A	0	1	N/A	0	0	1	1	0	0	0	1	8	Fair
Esaki et al.,	1	1	N/A	1	1	N/A	0	1	1	1	1	1	0	1	10	Good
Yen et al.,	1	1	N/A	0	1	N/A	0	1	1	1	1	1	0	1	9	Fair
Liu, et al.,	1	1	N/A	0	0	1	0	0	1	1	0	1	0	1	7	Fair
Llena et al.,	0	1	N/A	0	1	0	0	1	0	1	0	0	0	0	4	Poor
Linden et al.,	1	1	1	1	1	1	0	1	1	1	1	1	0	1	12	Good
Miley et al.,	1	1	N/A	1	1	1	0	1	1	1	1	1	0	1	11	Good
Chapple, et al.,	1	1	0	0	1	0	1	1	1	1	1	1	0	1	10	Good
Dietrich et al.,	1	1	N/A	N/A	1	N/A	1	1	1	1	1	1	N/A	1	10	Good
Erdemir & Bergstrom,	1	1	N/A	N/A	1	N/A	1	1	1	1	1	1	N/A	1	10	Good
Mdinaradze,	0	1	0	0	1	0	0	1	0	1	1	0	0	0	5	Poor
Lingstrom et al.,	1	1	0	0	1	1	1	1	1	1	1	1	0	1	11	Good
Neiva et al.,	1	1	0	0	1	1	0	1	0	1	1	0	0	1	8	Fair
Staudte et al.,	1	1	1	0	1	1	0	1	0	1	1	0	0	1	9	Fair
Dietrich et al.,	1	1	1	0	1	1	1	1	N/A	1	1	1	0	1	11	Good

Prasad et al.,	1	1	0	0	1	0	0	1	1	1	1	1	0	1	9	Fair
MacKeown et al.,	1	1	N/A	1	1	1	N/A	1	1	1	1	1	1	0	11	Good
Krall et al.,	1	1	0	0	1	1	1	1	1	1	1	1	0	1	11	Good
Al Malik et al.,	1	1	0	0	1	1	N/A	1	1	1	1	1	1	1	11	Good
Aine et al.,	1	1	0	1	1	0	1	1	1	1	1	1	1	1	12	Good
Petti et al.,	1	1	0	0	1	0	1	1	0	1	1	0	0	0	7	Fair

1 Was the research question or objective in this paper clearly stated?; 2 Was the study population clearly specified and defined?; 3 Was the participation rate of eligible persons at least 50%? ; 4 Were all the subjects selected or recruited from the same or similar populations (including the same time period)? Were inclusion and exclusion criteria for being in the study pre-specified and applied uniformly to all participants?; 5 Was a sample size justification, power description, or variance and effect estimates provided?; 6 For the analyses in this paper, were the exposure(s) of interest measured prior to the outcome(s) being measured?; 7 Was the timeframe sufficient so that one could reasonably expect to see an association between exposure and outcome if it existed?; 8 For exposures that can vary in amount or level, did the study examine different levels of the exposure as related to the outcome (e.g., categories of exposure, or exposure measured as continuous variable)?; 9 Were the exposure measures (independent variables) clearly defined, valid, reliable, and implemented consistently across all study participants?; 10 Was the exposure(s) assessed more than once over time?; 11 Were the outcome measures (dependent variables) clearly defined, valid, reliable, and implemented consistently across all study participants?; 12 Were the outcome assessors blinded to the exposure status of participants?; 13 Was loss to follow-up after baseline 20% or less?; 14 Were key potential confounding variables measured and adjusted statistically for their impact on the relationship between exposure(s) and outcome(s)?

Table S3. List of excluded papers after full evaluation.

Author	Year	Sources	Reason for exclusion
Kakade et al.,	2018	Int J Sports Physiol Perform 2008 Dec;3(4):516-30.	case study
Gutierrez et al.,	2018	Am J Case Rep 2018; 19: 392-396	case study
Dobros et al.,	2018	Quintessence International 2018, 49, 408-412	case study
Bryce & MacBeth	2014	J Royal Naval Medical Service 2014, 100, 328-332	case study
Thomson et al.,	2015	Head Neck 2015, 37:, 182-187,	different outcome (salivary cystic carcinoma)
Kantaputra et al.,	2013	Am J Med Genet Part A 2014, 164A, 1-9.	different outcome (genetic syndrome)
Palacios et al.,	2016	Caries Res 2016;50:560-570	not possible to determine vitamins role
Howell et al.,	2001	J Am Coll Cardiol 2001, 37, 445-5	non pertinent not possible to determine vitamins role
Shim et al.,	2018	Caries Res 2018, 52, 71-77	not possible to determine vitamins role
Wegehaupt et al.,	2016	Swiss Dent J 2016, 126, 457-465	in vitro study
Buajeeb et al.,	2008	Journal of Oral Science, 2008, 50, 461-7	not pertinent with the aim of the review
Souza et al.,	2013	J Appl Oral Sei 2013, 21, 601-6	not human study
Joshi et al.,	2004	Br J Oral Maxi Surg 2004 42, 299-306	not pertinent with the aim of the review
Mdnaradze	2006	Georg Med New 2006, 113, 60-63	non pertinent not possible to determine vitamins role
Oteri et al.,	2016	J Craniofac Surg 2016, 27: 469-476	not pertinent with the aim of the review
